# 12-month prevalence of known chronic obstructive pulmonary disease (COPD) in Germany

**DOI:** 10.17886/RKI-GBE-2017-065

**Published:** 2017-10-09

**Authors:** Henriette Steppuhn, Ronny Kuhnert, Christa Scheidt-Nave

**Affiliations:** Robert Koch Institute, Department of Epidemiology and Health Monitoring, Berlin

**Keywords:** CHRONIC OBSTRUCTIVE PULMONARY DISEASE (COPD), PREVALENCE, ADULTS, HEALTH MONITORING, GERMANY

## Abstract

Chronic obstructive pulmonary disease (COPD) is associated with a high disease burden and is one of the leading causes of death worldwide. Smoking is the key modifiable risk factor for COPD in Germany. GEDA 2014/2015-EHIS surveyed the 12-month prevalence of known COPD using the European indicator on self-reported chronic bronchitis, chronic obstructive pulmonary disease, emphysema. Among adults aged 18 years or older with complete information on the indicator (n=22,702), the 12-month prevalence of known COPD is 5.8% (5.8% for women and 5.7% for men). In both genders, the prevalence increases strongly with age. Overall, the presence of COPD was more often reported by women and men with a low educational level than by those with a higher one. In a comparison of federal states, the 12-month prevalence of known COPD varies between 3.6% and 7.5% for women and 4.3% and 11.2% for men.

## Introduction

Chronic obstructive pulmonary disease (COPD) is associated with a high disease burden and is one of the leading causes of death in Germany and globally [1-3]. COPD is a prevalent chronic disease of middle and older age [1, 4-7]. It is marked by chronic inflammation and progressive obstruction (narrowing) of the airways and destruction of lung tissue (parenchyma) [1, 8]. Chronic cough and phlegm production (chronic bronchitis) as well as a permanent over-inflation of the air sacks (emphysema) are common among COPD patients and often occur together [1, 8]. Moreover, shortness of breath under physical strain is a typical symptom. At more progressed stages of the disease, patients may also suffer from shortness of breath even at rest [1, 8].

Smoking is the most important modifiable risk factor for COPD in Germany [1, 7-9]. The risk of developing COPD is thereby related to the total amount of cigarette smoking over time (pack years) [7, 9]. Moreover, specific occupational exposures (e.g., coal dust) are important COPD risk factors [1, 7, 9, 10]. COPD is therefore considered a potentially preventable disease [1, 8]. However, impaired growth and functional development of the lungs also affect a person’s COPD risk [1, 9, 11]. Besides genetic factors and prenatal influences (e.g., maternal smoking during pregnancy), repeated respiratory infections in early childhood, exposure to airborne pollutants, or childhood asthma potentially contribute to developing COPD in later life [1, 7, 9, 12, 13].

**GEDA 2014/2015-EHIS**

**Data holder:** Robert Koch Institute

**Aims:** To provide reliable information about the population’s health status, health-related behaviour and health care in Germany, with the possibility of a European comparison

**Method:** Questionnaires completed on paper or online

**Population:** People aged 18 years and above with permanent residency in Germany

**Sampling:** Registry office sample; randomly selected individuals from 301 communities in Germany were invited to participate

**Participants:** 24,016 people (13,144 women; 10,872 men)

**Response rate:** 26.9%

**Study period:** November 2014 - July 2015

**Data protection:** This study was undertaken in strict accordance with the data protection regulations set out in the German Federal Data Protection Act and was approved by the German Federal Commissioner for Data Protection and Freedom of Information. Participation in the study was voluntary. The participants were fully informed about the study’s aims and content, and about data protection. All participants provided written informed consent.

More information in German is available at

www.geda-studie.de

Adults with COPD frequently suffer from other chronic conditions such as cardiovascular diseases [1, 7, 9]. These concurrent conditions considerably impact the quality of life of COPD patients and contribute to the high COPD-related costs of illness [14-17]. COPD-related costs of illness are also significantly determined by the severity of the disease [17, 18]. A recent study of COPD patients in Germany revealed that in comparison to a control group from the region of Augsburg, excess (direct) costs resulting from healthcare utilization ranged between 2,595 and 8,924 EUR per patient in 2012 [17]. The indirect costs (e.g., due to work absence) were significantly higher ranging from 8,621 to 27,658 EUR [17].

## Indicator

GEDA 2014/2015-EHIS surveyed the prevalence of known COPD during the past 12 months based on an instrument from the indicator set of the European health monitoring by using self-administered paper-based or online questionnaires. Respondents were asked, 'During the past 12 months, have you had any of the following diseases or conditions?' This question was followed by a list of conditions that also included 'chronic bronchitis, chronic obstructive pulmonary disease, emphysema'. Out of a total of 24,016 respondents aged 18 years or older (13,144 women, 10,872 men), 1,314 respondents (696 women and 618 men) with missing information on the indicator were excluded from the analysis. COPD prevalence was calculated using a weighting factor that corrects for deviations within the sample from the German population structure (as of 31 December 2014) with regard to gender, age, district type and education. The district type reflects the degree of urbanisation and corresponds to the regional distribution in Germany. The International Standard Classification of Education (ISCED) was used to classify the responses provided on educational level [19]. Lange et al. [20] set out the details of the methodology applied in GEDA 2014/15-EHIS including a description of the method used to calculate the weighting factor and an assessment of the response rate. Background information on GEDA 2014/15-EHIS are also provided in the article German Health Update: New data for Germany and Europe, which was published in Issue 1/2017 of the Journal of Health Monitoring.

## Results and discussion

**Figure 1:**
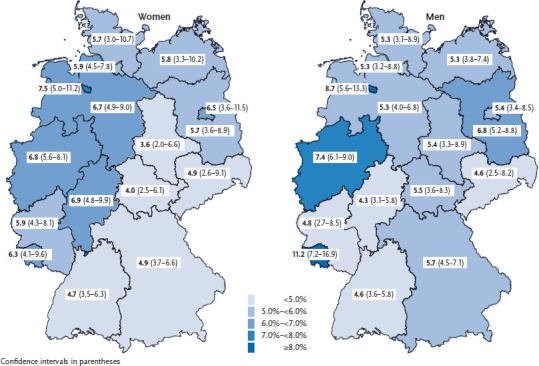
12-month prevalence of known COPD according to gender and federal state (n=12,448 women; n=10,254 men) Source: GEDA 2014/2015-EHIS

In GEDA 2014/2015-EHIS, 5.8% of adults aged 18 years or older reported the presence of COPD during the past 12 months. The 12-month prevalence of known COPD for women (5.8%) is comparable to the prevalence for men (5.7%). In both genders, the 12-month prevalence increases strongly with age (Table 1). Overall, the presence of COPD was more often reported by women and men with a low educational level than by those with a medium or high one (8.1% vs. 5.7% and 4.0%). Stratified by age and sex, these differences with regard to educational level are particularly evident for women under 65 years of age and for men aged 45 to 64 years (Table 1). The prevalence of known COPD varies considerably between federal states, ranging from 3.6% in Saxony-Anhalt to 7.5% in Bremen for women and from 4.3% in Hesse to 11.2% in Saarland for men (Figure 1).

When comparing these results on the prevalence of known COPD with the results from previous epidemiological studies, considerable methodological differences need to be taken into account. For example, a comparison with the results from the interview survey of adults aged 18 years or older conducted by the Robert Koch Institute in 2012 (GEDA 2012) is not possible because both the type of the interview (written/online questionnaire now, telephone interview then) and the indicator differ [21]. GEDA 2012 surveyed the 12-month prevalence of physician-diagnosed chronic bronchitis defined as coughing with phlegm for at least 3 months per year [21]. Prevalence estimates for this indicator were 6.0% for women and 4.0% for men [21].

Besides interview data, COPD prevalence estimates are mainly based on data obtained from a pulmonary function test (spirometry) [4, 6, 18, 22, 23]. However, there is poor agreement between estimates of spirometrically defined and self-reported COPD prevalence [4, 6, 22, 24, 25]. For example in a population-based cohort study among adults 41 to 90 years of age from the region of Augsburg (KORA), less than 40% of participants with spirometrically defined COPD reported to have physician-diagnosed COPD [22]. This was explained by a high number of undiagnosed COPD cases [22], a fact that results in an underestimation of the actual COPD prevalence when using self-reported information [1, 9, 25, 26].

In line with other studies, GEDA 2014/2015-EHIS survey data also demonstrate that COPD is an age-associated disease [1, 4-7]. A comparison with prevalence estimates of spirometrically defined COPD must, however, take into account that lung function generally decreases with age and also shows considerable variation in the older population [27-29]. In particular, age-specific estimates on COPD prevalence based on spirometry data differ depending on the chosen reference criteria and method of examination, e.g. with or without the use of medications to dilate airways [1, 9, 22, 27-29]. Besides, the available prevalence data on spirometrically defined COPD is based alone on the detection of an airflow obstruction [1, 4, 6]. A clinical diagnosis of COPD, however, also requires the consideration of risk factors, symptoms, the corresponding individual clinical history and, where necessary, further clinical examinations [1, 9, 27, 29].

Results from international surveys including data from Germany reveal a higher prevalence of spirometrically defined COPD among men than among women [5, 7, 18, 24, 30, 31]. For example, the estimated prevalence of spirometrically defined COPD among adults 40 year of age or older was 9.3% for women and 18.1% for men based on data collected in the German study centre Hanover of the international Burden of Obstructive Lung Disease (BOLD) study in 2006 [24]. This difference between women and men was observed independent of disease severity [5]. Higher prevalence among men, however, was only seen among those aged 50 years or older and was considered to be potentially related to the gender-specific differences in smoking habits across age groups [24]. In agreement with the findings presented here, results from this previous survey demonstrated that there were no gender differences regarding the prevalence of a known COPD: 7.7% of women and 7.6% of men reported to have been diagnosed with COPD by a physician [24]. Further analyses on the basis of BOLD and other survey data indicated underlying differences in COPD awareness between women and men, as male gender was associated with an undiagnosed COPD [25]. In the GEDA 2014/2015-EHIS survey conducted across Europe, data collection on the prevalence of known COPD was based on the composite indicator compelling information on chronic bronchitis, chronic obstructive pulmonary disease, emphysema in line with other cross-country surveys [4, 6, 23]. Thereby, however, adults with chronic cough and phlegm production might have been included who have otherwise normal spirometry results and will not develop COPD in later life [1, 32-34]. This is particularly relevant with regard to the prevalence of known COPD assessed among young adults in GEDA 2014/2015-EHIS [31]. Moreover, current findings on the prevalence of known COPD are based on the self-assessment of respondents and not on self-reported medical diagnoses [4, 6, 22, 24]. This increases the likelihood of misclassifying patients with other diseases that are marked by similar symptoms, in particular asthma [31].

In accordance with other data on spirometrically defined COPD, GEDA 2014/2015-EHIS results indicate a higher prevalence of known COPD among adults with a low educational level compared to those with a higher one [7, 9, 35, 36]. In line with these findings, comparable differences in prevalence had previously been reported with regard to different socio-economic criteria [7, 9, 35, 36]. GEDA 2014/2015-EHIS revealed considerable regional differences in the prevalence of known COPD not only between federal states but also between EU countries. Germany thereby ranked in the group of countries presenting the highest prevalence [37] (see issue 1/2017 Journal of Health Monitoring). The interpretation of social and regional differences in known COPD prevalence needs to consider differences in the distribution of risk factors such as smoking (see also the Fact sheet Smoking among adults in Germany in issue 2/2017 Journal of Health Monitoring) as well as in COPD awareness and care provision [36, 38-40]. Periodically repeated collection of nationally representative population-based data on major modifiable risk factors as well as on lung function, diagnosis, symptoms, and mortality of COPD is essential in order to identify successes and remaining or new challenges of COPD prevention and care.

## Key statements

- The 12-month prevalence of known COPD is 5.8% for women and 5.7% for men.
- In both genders, the 12-month prevalence of known COPD increases strongly with age.
- The presence of COPD was more often reported by women and men with a low educational level than by those with a higher one.

## Figures and Tables

**Table 1:** 12-month prevalence of known COPD according to gender, age and educational level (n=12,448 women; n=10,254 men) Source: GEDA 2014/2015-EHIS

Women	%	(95% CI)	Men	%	(95% CI)
**Women total**	**5.8**	**(5.2-6.4)**	**Men total**	**5.7**	**(5.2-6.3)**
**18-29 Years**	2.4	(1.6-3.5)	**18-29 Years**	1.3	(0.8-2.3)
Low education	4.5	(2.5-8.2)	Low education	2.0	(0.7-5.3)
Medium education	2.0	(1.2-3.3)	Medium education	1.2	(0.6-2.4)
High education	0.7	(0.3-1.9)	High education	0.7	(0.3-1.8)
**30-44 Years**	3.4	(2.7-4.4)	**30-44 Years**	2.0	(1.4-2.9)
Low education	7.4	(4.5-11.9)	Low education	2.8	(1.2-6.2)
Medium education	2.9	(2.0-4.1)	Medium education	2.5	(1.7-3.9)
High education	2.5	(1.5-4.0)	High education	0.9	(0.4-1.9)
**45-64 Years**	5.1	(4.3-5.9)	**45-64 Years**	6.3	(5.4-7.4)
Low education	7.2	(5.2-9.8)	Low education	12.1	(8.8-16.3)
Medium education	5.1	(4.2-6.2)	Medium education	7.0	(5.8-8.5)
High education	3.3	(2.4-4.5)	High education	3.1	(2.4-4.1)
**≥ 65 Years**	11.0	(9.5-12.7)	**≥ 65 Years**	12.5	(10.9-14.3)
Low education	10.8	(8.6-13.5)	Low education	15.5	(11.7-20.1)
Medium education	11.2	(9.2-13.6)	Medium education	12.2	(10.0-14.8)
High education	9.4	(6.1-14.0)	High education	11.6	(9.4-14.3)
**Total (women and men)**	**5.8**	**(5.4-6.2)**	**Total (women and men)**	**5.8**	**(5.4-6.2)**

CI=Confidence interval

* n=50 additional missing values (25 women and men) when stratifying by educational level
